# Grid computing in image analysis

**DOI:** 10.1186/1746-1596-6-S1-S12

**Published:** 2011-03-30

**Authors:** Klaus Kayser, Jürgen Görtler, Stephan Borkenfeld, Gian Kayser

**Affiliations:** 1 UICC-TPCC, Charite, Berlin, Germany; 2 IAT, Heidelberg, Germany; 3 IBM, Mainz, Germany; 4 Institute of Pathology, University Freiburg, Freiburg, Germany

## Abstract

Diagnostic surgical pathology or tissue–based diagnosis still remains the most reliable and specific diagnostic medical procedure. The development of whole slide scanners permits the creation of virtual slides and to work on so-called virtual microscopes. In addition to interactive work on virtual slides approaches have been reported that introduce automated virtual microscopy, which is composed of several tools focusing on quite different tasks. These include evaluation of image quality and image standardization, analysis of potential useful thresholds for object detection and identification (segmentation), dynamic segmentation procedures, adjustable magnification to optimize feature extraction, and texture analysis including image transformation and evaluation of elementary primitives.

Grid technology seems to possess all features to efficiently target and control the specific tasks of image information and detection in order to obtain a detailed and accurate diagnosis.

Grid technology is based upon so-called nodes that are linked together and share certain communication rules in using open standards. Their number and functionality can vary according to the needs of a specific user at a given point in time. When implementing automated virtual microscopy with Grid technology, all of the five different Grid functions have to be taken into account, namely 1) computation services, 2) data services, 3) application services, 4) information services, and 5) knowledge services.

Although all mandatory tools of automated virtual microscopy can be implemented in a closed or standardized open system, Grid technology offers a new dimension to acquire, detect, classify, and distribute medical image information, and to assure quality in tissue–based diagnosis.

## Introduction

The implementation of digital or computational pathology in routine diagnostic surgical pathology or tissue - based diagnosis has already started, and seems to be a progressive and accelerating process [1-5]. Numerous institutes of pathology have installed commercially available scanning systems despite the relatively high investment [6,7]. They mainly use the systems for clinical – pathological conferences and for educational purposes [8]. A few institutes of pathology have already implemented the scanning systems into their routine work, mainly in parallel application to conventional microscopy [9]. In addition to this development industry sponsored investigations focus on so-called automated virtual microscopy, which is the computerized support of the pathologist’s diagnostic work at different levels [4]. Such a system would require a series of tools in order to reach the final aim of automated slide pre-screening or even diagnostic suggestion [10]. These different tools have not necessarily to run on an individual or even the same machine. A distributed network using specific standardized communication paths might have advantages in terms of velocity, control of work load, quality assurance, and continuous technical development and expansion [11]. One technological solution of such a system is the so-called Grid, which is in principle a network of computers that communicate and control each other using standardized software. The specific tasks and mandatory conditions how to implement an automated virtual microscope in a Grid are herein described.

## Definition and description of automated virtual microscopy

Automated virtual microscopy is the diagnostic work of a pathologist using a fully automated digital (virtual) microscope [12]. Dependent upon the level of automation different so – called microscope assistants can be defined. These include

1. Image standardization and quality assessment

2. Crude image analysis for potential segmentation features

3. Evaluation of image primitives (elementary events)

4. Selection of fields of view (regions of interest (ROI))

5. Segmentation of biological meaningful objects

6. Computation of structures

7. Image transformation and texture analysis

8. Classification of obtained image data into diagnoses

9. Evaluation of diagnostic accuracy and consistency

10. Final report and feedback to potential additional laboratory data (images)

11. Restart and refinement of image information and diagnosis (additional stains, etc.).

The listed series of virtual microscope assistants can be extended by additional tools such as image data banks, automated notification of images, links and retrieval in public data banks, or expert and control consultations. The required computation power differs between the listed tools remarkably: Evaluation of image primitives, selection of regions of interest, and segmentation of biological meaningful objects require intensive computation, image standardization and disease classification less intensive computation, and final reporting and restart the least one [12].

Some of these tools are already implemented in an internet based, automated image analysis system for immunohistochemical images (EAMUS), accessible via http://WWW.DIAGNOMX.EU. This system can be considered as a very simple arrangement of a Grid [13].

## Definition and description of Grid technology

Basically, a Grid is an Internet embedded network consisting of a broad variety of connected nodes which correspond to servers. They serve as platform of communication standards, and permit the users to concentrate solely on their individual tasks. In addition to the necessary communication standards a Grid provides also network computing, i.e. distributed computing of the user’s tasks. Thus, it is a derivative of the development and maturation of the Internet [14]. In analogue to the implementation of power supply “grids” that continuously supply households with electrical power independently where the power has been generated a Grid assures standardized information transfer between different nodes. The user has not to care about nodes whether they are data sources, image servers, or highly specialized measuring systems. Similar to telephone services the user is not informed about the various embedded communication pathways (e.g. cable, microwave, satellite), and to which computers he actually is connected to. They might be located in the Far East, in Europe, or in the USA. These approaches to network computing are also called metacomputing, scalable computing, global computing, and Internet computing. The main applications include large-scale computational and data intensive problems in science, engineering, and commerce [11].

## Components of Grid technology

A Grid consists of a set of connected computers that can act as the end users or clients, as managers to distribute and control the wanted tasks (so called distribution and control nodes) and as computation machines. In other words, a Grid is a network of computers, anyone able to perform the requested tasks. Therefore, we need at least four different types of programs (layers) in a Grid:

1. Data input and output programs (image acquisition and presentation)

2. Application programs (image standardization, evaluation of information, etc.)

3. Communication programs (Web communication standards, server access, etc.)

4. Network management programs (workload of computers, task performance control, etc.)

The listed programs belong to different program layers that are of hierarchical order, starting with front – ends (data input, display) and finally positioned at the network control and management respectively. The backbone of the Grid infrastructure is a computer–based collaborative environment using a management software layer (Middleware). This software layer again works in a distributed manner, and requires its own computation nodes, the so – called brokers. The brokers administer the workload and potential problems, discover free resources, and control the processing of the end user tasks.

## Grid services

The described infrastructure of a Grid has been designed for a broad variety of services that can be grouped into five different aims:

*Computational services* have been described as first applications of a Grid. They solve tasks that require high computational power, for example to solve recursive formulas. They are in use for of high energy experiments, or astrophysics. In its simplest manner, one (or several) of the distributed supercomputers take the computational task as long as they are not busy with or overloaded by other tasks. Once this happens, the task and its computational stage are transferred to other included supercomputers, etc. as long as the task is not finished. A priority set of different tasks can stop the computation of an individual task and save its present stage as long as other, more important tasks have not been finished. Examples of computational Grids include: NASA IPG [15], the World Wide Grid (Buyya R. The World-Wide Grid. http://www.buyya.com/ecogrid/wwg/) [16], and the NSF Tera-Grid. (http://www.teraGrid.org/) [17]. Computational services would be appropriate for detection of regions of interest, image segmentation and object identification tools as well as for image comparison (block comparison) [5].

*Data services* are implemented in several search machines, and offer secure access to distributed datasets. They manage all functions that are used in conventional libraries such as data access, retrieval, storage, replication, or search for data in catalogues of individual or distributed libraries. A more simple structure has been implemented by so-called links, or data-Grids, that are used in the area of high-energy physics [18,19] or drug design [16]. http://www.buyya.com/vlab/. Data services would be appropriate to set up classification of diseases, image labeling, or identification of objects, structures, and textures in virtual microscopy.

*Application services* represent the next higher level and give access to remote software, libraries and Web services. They provide the adequate formulas to be applied on implemented data sets, for example a databank of parameters etc. to fulfill this task. In tissue–based diagnosis, the EAMUS™ [12,13,20] can be considered as a simple, one node implementation of this service. A well known Grid application service is, for example, created by NetSolve [21]. In virtual microscopy several tasks could be performed with application services, especially diagnosis oriented computations of image standardization, features, and regions of interest.

*Information services* are at an advanced level of application services. They put into relationship data of computational information, and/or application services and present the obtained information. In virtual microscopy, a simple implementation could be created by combining image measurements (for example provided by EAMUS™ services) with an existing telepathology information system such as UICC-TPCC, or iPATH. Another more common example of low-level information services are Meta Data, i.e. a context oriented manner to present, store, access, share, and maintain information. Information services provides also the EU–sponsored Virolab Grid, a project that addresses the problem of HIV drug resistance and offers the integration of biomedical information, advanced applications, patients’ data, and intelligent literature access (http://www.gridwisetech.com/virolab).

*Knowledge services* are the most advanced Grid services from the viewpoint of informatics. They are designed to improving with the algorithms of acquiring, using, retrieving, publishing, or maintaining knowledge. Knowledge is considered as information applied to achieve a goal, solve a problem, or execute a decision. A characteristic example is data mining for automatically building a new knowledge. In virtual microscopy it would be an appropriate tool in automated screening and analyzing virtual slides prior to be viewed by the pathologists, or to automatically inform the pathology laboratory about additional investigations in data needed to evaluate a definite diagnosis (immunohistochemical stains, gene analysis, etc.) [8,22-25].

## Perspectives

Grids are rarely found in the medical work until today [11]. The amount of data to be handled and applicable in medical diagnosis and treatment and the required computational power are small in comparison to those needed in natural sciences, for example in astrophysics or molecular modelling [11]. The scenario will probably change once the image generation and analysis procedures have fully been digitized [6,7,25,26]. The digitalization of functional data such as ECG (electrocardiograms) will probably not require to be implemented in network computing systems that offer high speed computing of huge amounts of data in contrast to diagnostic image systems in radiology and pathology. A fully automated virtual microscopy system has to work with image data that count to Terabytes [5,7,24]. These data have to be acquired, transported, stored, retrieved, and analyzed. Sophisticated image compression, construction of the necessary logistics, and data selection are methods to significantly reduce the amount of data [8]. To our opinion, they seem to be more a help construction than a found solution. Intelligent distribution into several cooperative hands of an otherwise not or only difficult to handle task has always been a successful method, for both man and animals lining in social communities.

Grid technology offers a robust and firm framework to fulfil the requirements of digitized diagnostic surgical pathology [7,25]. The needed computations of the acquired digital images of whole glass slides, the original size of the images, and the medical requirements of image presentation and display seem to exactly meet the formal framework of a Grid [11]. This is even more obvious, if the medical environment such as hospital information system, embedding in an open standardized communication system (expert consultation), or tasks of medical education and research are taken into account.

In its final consolidation automated virtual microscopy would require a Grid that offers all known services, starting from computational services and reaching the level of knowledge services. Computational services and data services would provide the pathologist with information sources to be interpreted and evaluated still interactively [5,27,28]. The next two levels of services (application and information services) would provide diagnostic assistants that are still controlled visually and interactively by the pathologist. The final stage of implementing knowledge services would finally result in an automated diagnostic system which will serve as new diagnostic quality level to improving the details of diagnosis and associated treatment.

## Competing interests

The authors declare that they have no competing interests.

## References

[B1] Bueno G Image processing methods and architectures in diagnostic pathology Folia Histochem Cytobiol 2009 47 4 691 7 10.2478/v10042-009-0116-x 20430740

[B2] Cnudde V Virtual histology by means of high-resolution X-ray CT J Microsc 2008 232 3 476 85 10.1111/j.1365-2818.2008.02142.x 19094024

[B3] Gilbertson J Yagi Y Histology, imaging and new diagnostic work-flows in pathology Diagnostic Pathology 2008 3 Suppl 1 S14 10.1186/1746-1596-3-S1-S14 18673502 PMC2500122

[B4] Kayser K Kayser G R.O. J. Gu Virtual Microscopy and Automated Diagnosis Virtual Microscopy and Virtual Slides in Teaching, Diagnosis and Research 2005 Taylor & Francis: Boca Raton

[B5] Kayser K Molnar B Weinstein RS Virtual Microscopy - Fundamentals - Applications - Perspectives of Electronic Tissue - based Diagnosis 2006 VSV Interdisciplinary Medical Publishing

[B6] Lundin M A European network for virtual microscopy--design, implementation and evaluation of performance Virchows Arch 2009 454 4 421 9 10.1007/s00428-009-0749-3 19280223

[B7] Marchevsky AM The use of virtual microscopy for proficiency testing in gynecologic cytopathology: a feasibility study using ScanScope Arch Pathol Lab Med 2006 130 3 349 55 16519563 10.5858/2006-130-349-TUOVMF

[B8] Mori I Issues for application of virtual microscopy to cytoscreening, perspectives based on questionnaire to Japanese cytotechnologists Diagn Pathol 2008 3 Suppl 1 S15 10.1186/1746-1596-3-S1-S15 18673503 PMC2500096

[B9] Merk M Knuechel R Perez-Bouza A Web-based virtual microscopy at the RWTH Aachen University: Didactic concept, methods and analysis of acceptance by the students Ann Anat 20363112 10.1016/j.aanat.2010.01.008

[B10] Kayser K Theory of sampling and its application in tissue based diagnosis Diagn Pathol 2009 4 6 10.1186/1746-1596-4-6 19220904 PMC2649041

[B11] Gortler J Grid technology in tissue-based diagnosis: fundamentals and potential developments Diagn Pathol 2006 1 23 10.1186/1746-1596-1-23 16930477 PMC1564417

[B12] Kayser K AI (artificial intelligence) in histopathology--from image analysis to automated diagnosis Folia Histochem Cytobiol 2009 47 3 355 61 10.2478/v10042-009-0087-y 20164018

[B13] Kayser G Theory and implementation of an electronic, automated measurement system for images obtained from immunohistochemically stained slides Anal Quant Cytol Histol 2006 28 1 27 38 16566277

[B14] Oliveira IC Grid requirements for the integration of biomedical information resources for health applications Methods Inf Med 2005 44 2 161 7 15924167

[B15] Johnston W Gannon D Nitzberg B Grids as production computing environments: The engineering aspects of NASA's information power Grid Eighth IEEE International Symposium on High Performance Distributed Computing 1999 Redondo Beach, CA: IEEE Computer Society Press, Los Alamitos, CA

[B16] Buyya R The World-Wide Grid 2006 http://www.buyya.com/ecogrid/wwg/

[B17] NSF, T.-G. NSF Tera-Grid 2006 [cited

[B18] Cerello P GPCALMA: a Grid-based tool for mammographic screening Methods Inf Med 2005 44 2 244 8 15924184

[B19] Pacios LF Fernandez A CheckDen, a program to compute quantum molecular properties on spatial grids J Mol Graph Model 2009 28 2 102 12 10.1016/j.jmgm.2009.04.008 19447056

[B20] Kayser K Texture- and object-related automated information analysis in histological still images of various organs Anal Quant Cytol Histol 2008 30 6 323 35 19160697

[B21] Thomason MG Simulation of emission tomography using grid middleware for distributed computing Comput Methods Programs Biomed 2004 75 3 251 8 10.1016/j.cmpb.2004.02.003 15265623

[B22] Kayser K Towards an automated virtual slide screening: theoretical considerations and practical experiences of automated tissue-based virtual diagnosis to be implemented in the Internet Diagn Pathol 2006 1 10 10.1186/1746-1596-1-10 16764733 PMC1524814

[B23] Kayser K Digitized pathology: theory and experiences in automated tissue-based virtual diagnosis Rom J Morphol Embryol 2006 47 1 21 8 16838053

[B24] Weinstein RS Innovations in medical imaging and virtual microscopy Hum Pathol 2005 36 4 317 9 10.1016/j.humpath.2005.03.007 15891989

[B25] Yang L Virtual microscopy and grid-enabled decision support for large-scale analysis of imaged pathology specimens IEEE Trans Inf Technol Biomed 2009 13 4 636 44 10.1109/TITB.2009.2020159 19369162 PMC3683401

[B26] Oger M Automated region of interest retrieval and classification using spectral analysis Diagnostic Pathology 2008 3 Suppl 1 S17 10.1186/1746-1596-3-S1-S17 18673505 PMC2500116

[B27] Giansanti D Virtual microscopy and digital cytology: state of the art Ann Ist Super Sanita 46 2 115 22 20567061 10.4415/ANN_10_02_03

[B28] Johansen MV Towards Improved Diagnosis of Zoonotic Trematode Infections in Southeast Asia Adv Parasitol 73C 171 195 10.1016/S0065-308X(10)73007-4 20627143

